# Development and validation of the Socioeconomic Status Composite Scale (SES-C)

**DOI:** 10.1186/s12889-023-16531-9

**Published:** 2023-08-24

**Authors:** Hala Sacre, Chadia Haddad, Aline Hajj, Rony M. Zeenny, Marwan Akel, Pascale Salameh

**Affiliations:** 1 Institut National de Santé Publique, d’Épidémiologie Clinique Et de Toxicologie-Liban (INSPECT-LB), Beirut, Lebanon; 2 School of Medicine, Lebanese American University, Byblos, Lebanon; 3 Research Department, Psychiatric Hospital of the Cross, P.O. Box 60096, Jal Eddib, Lebanon; 4 School of Health Sciences, Modern University for Business and Science, Beirut, Lebanon; 5 Laboratoire de Pharmacologie, Pharmacie Clinique Et Contrôle de Qualité Des Médicament (LPCQM), Faculty of Pharmacy, Saint Joseph University of Beirut, Beirut, Lebanon; 6 Faculté de Pharmacie, Université Laval, Québec, Canada; 7 Oncology Division, CHU de Québec Université Laval Research Center, Québec, Canada; 8 Department of Pharmacy, American University of Beirut Medical Center, Beirut, Lebanon; 9 School of Pharmacy, Lebanese International University, Beirut, Lebanon; 10 School of Education, Lebanese International University, Beirut, Lebanon; 11 Faculty of Pharmacy, Lebanese University, Hadat, Lebanon; 12 Department of Primary Care and Population Health, University of Nicosia Medical School, 2417 Nicosia, Cyprus

**Keywords:** Socioeconomic status, Financial distress, Validation, Assessment, Composite scale, Developing country

## Abstract

**Background:**

Socioeconomic status (SES) is a critical multifactorial determinant of health and plays a significant role in shaping an individual’s health outcomes. While a composite scale has been proposed to measure SES in children, to our knowledge, limited composite scales were developed for adults in different contexts, highlighting the need for a comprehensive and valid SES measure to elucidate the relationship between SES and health in this population.

**Objective:**

This study aimed to develop and validate a composite scale that measures the socioeconomic status in Lebanon and assess its correlates in a socioeconomic crisis context.

**Methods:**

An online study was carried out between October and November 2022 across all Lebanese regions. Snowball sampling was used to enroll 448 adults living in Lebanon through a questionnaire created on Google Forms and shared by WhatsApp to a first sample from all geographic areas.

**Results:**

The developed composite scale (SES-C) was found to be reliable and valid. It was based on several aspects of socioeconomic status, i.e., participant education level, family head education level, perceived social class, not being in debt, not receiving financial help, crowding index, participant work status, family head work status, monthly household income, and financial well-being. Furthermore, high SES was significantly associated with married status, older age, alcohol consumption, the absence of chronic disease, easy access to healthcare, private insurance coverage, and the number of rooms in the house in the bivariate analysis. In the multivariable analysis, high SES was significantly associated with age (ORa-1.13; *p* = 0.011) and easy access to healthcare (ORa = 7.81; *p* = 0.001) and inversely associated with chronic disease (ORa = 0.17; *p* = 0.002). Similar results with lower magnitude were found for moderate SES.

**Conclusion:**

The study successfully developed and validated a composite scale (SES-C) for measuring the socioeconomic status in Lebanon, taking into account the complexities of the Lebanese context. The scale was found to be reliable and valid, and its results showed significant correlations with various factors such as older age, lower risk of chronic disease, and easy access to healthcare.

## Background

Socioeconomic status (SES) is a complex and multifaceted concept that encompasses multiple dimensions, including income, education, occupation, and social and economic status [1, 2]. It can be defined as the status of a person or a household within a community [3, 4] and is widely recognized as a critical determinant of an individual’s health and well-being, influencing access to resources and opportunities and contributing to health disparities [5].

Research has shown that individuals with lower SES are more likely to experience poorer health outcomes, such as the increased risk of chronic diseases [6] and reduced life expectancy [7]. This relationship between SES and health outcomes is well documented and consistent across populations, regardless of cultural or geographical differences [6]. Hence, social and economic inequalities in a country are a significant public health concern [8]. Despite the recognition of the importance of SES in shaping health outcomes, there is currently a limited number of comprehensive and valid measures of SES for adults: An Iranian study addressed the issue qualitatively [9], while, in Sweden, research recommended using composite tools to study health inequities in older adults [10]. The latter tool cannot be used in developing countries, where lifestyle and health systems are different. To our knowledge, no composite scale has been developed for adults, highlighting the need for a valid and reliable measure of SES that can capture the relationship between SES and health in adult populations, particularly in developing countries. Such a tool would also provide valuable information for policymakers, health professionals, and researchers [11] and inform evidence-based interventions to reduce health disparities and improve the health of populations [12].

Measuring SES is a challenge for several reasons, such as its multidimensional aspect and the absence of a standardized method to assess it, which can yield varying results, as documented by a systematic review [13]. Furthermore, SES is not a static concept; it fluctuates over time, creating the need for ongoing, accurate measurement, which can be challenging to achieve in practice. Indeed, discrepancies have long been described, and the problem remains unsolved due to the multiple measuring tools [14], particularly in developing countries [15]. According to the American Psychological Association (APA), three levels should be considered when measuring SES, i.e., the societal level, the community or neighborhood level, and the individual level [16]. The APA also recommends assessing education, income, employment, and family size/relationships to measure SES [16]. A recent study has reviewed the value of three widely used scales, i.e., the Kuppuswamy, BG Prasad, and Udai Pareekh scales, and found limitations to their use in rural and urban settings simultaneously [17]. Therefore, composite scales can help assess SES, considering its multiple facets. A composite SES scale has already been suggested for children; however, it cannot be used among adults as its variables are child-specific [12].

In Lebanon, a developing Middle Eastern country, the severe socioeconomic crisis increased poverty [18] and significantly affected the health sector, leading to a decline in healthcare services [19], particularly among vulnerable populations [20]. Indeed, the political turmoil, COVID-19, and the Beirut port blast in August 2020 resulted in severe socioeconomic and health crises and the steep depreciation of the local currency, with disastrous consequences on the health sector, with public health insurance entities and patients no longer being able to afford the actual cost of healthcare [21]. Thus, developing a scale that accurately measures SES in challenging contexts is crucial to identify vulnerable populations and guide interventions. Therefore, this study aimed to develop and validate a composite scale that measures the socioeconomic status in Lebanon and assess its correlates in a socioeconomic crisis context.

## Methods

### Study design

This online study was carried out between October and November 2022 across all Lebanese regions during the socioeconomic crisis. Snowball sampling was used to enroll participants through a questionnaire created on Google Forms and shared by WhatsApp to a first sample from all geographic areas (Beirut, Beqaa, Mount Lebanon, South, and North). This first sample is constituted of one family member or friend of pharmacy students from the Lebanese International University (*n* = 100 students), distributed all over the Lebanese territory; the respondent was then encouraged by the student to send the survey link to 10 of their acquaintances. We expected 50% response rate through this process, based on previous experience.

Respondents were briefed about the topic and the different aspects of the study at the beginning of the questionnaire. All people 18 years and above living in Lebanon were eligible to participate.

### Ethical aspect

The Research and Ethics committee at the Lebanese International University School of Pharmacy approved the study protocol (2022RC-041-LIUSOP). Consent was obtained from each participant at the beginning of the questionnaire. Anonymity and confidentiality were guaranteed across the data collection process.

### Sample size calculation

The minimum sample size was calculated using the G-Power software, version 3.0.10, considering the calculated effect size of 0.0526 and expecting a squared multiple-correlation coefficient of 0.05 (R^2^ deviation from 0) related to the Omnibus test of multiple regression. The minimum necessary sample was *n* = 415, assuming an alpha error of 5%, a power of 80%, and allowing for 20 predictors to be included in the model. A sample of 448 was collected when the decision was taken to stop the data collection process.

### Questionnaire and variables

The questionnaire was in Arabic and English, Arabic being the official language in Lebanon and the mother tongue of all respondents and English being widely spoken in the country. The questionnaire comprised two sections, including participants’ sociodemographic characteristics and validated scales.

In the first part of the questionnaire, participants reported their age, gender, nationality, area of residence (Lebanese governorate), achieved education level (of the participant and the head of the family; classified into illiterate, school, and university levels), work status (of the participant and head of the family), income, marital status, height, weight, number of children, cigarette and nargileh smoking, alcohol consumption, health status/diseases, healthcare access and coverage, and information about the household crowding index (calculated by dividing the number of people living in the house by the number of rooms, apart from the kitchen and bathrooms).

The second part of the questionnaire consisted of the following scales:

#### The InCharge Financial Distress-Financial Well-Being Scale (IFDFW)

IFDFW is a previously validated eight-item subjective measure developed in the United Kingdom to assess financial distress/well-being. Responses are reported on a scale ranging from 1 to 10, with higher scores indicating better financial well-being, and calculated by summing the answers to the eight questions (minimum of 8 and maximum of 80) [22]. Since this scale has been used but never validated in Lebanon, the first step was to assess its validity in the current sample before using it.

The scale was translated and back-translated for content validity confirmation; its construct validity was then assessed, and its convergent validity and reliability were confirmed. A pharmacist and medical editor, who is a senior research member, evaluated and verified the translation (from English into Arabic) performed by one of the collaborators while developing the survey tool. The IFDFW was then back-translated by a health professional from outside the research team. No discrepancies were noted during the process. The final questionnaire was piloted on bilingual participants to assess the clarity of questions. The total score was then divided into four interval categories (8–25; 26–50; 51–75; > 75 points) to facilitate the use of the scale, regardless of the corresponding numbers of individuals per category. This distribution is anticipated to more accurately reflect the worldwide distribution of middle and extreme socioeconomic classes [23], thus facilitating its application in global research settings.

#### The Socioeconomic Status Composite Scale (SES-C)

The composite scale was constructed using items related to well-being (IFDFW), income, external aids, debt magnitude, social class, crowding index, and education and work status of the participant and the head of the family, as recommended by the APA [16]. Moreover, as stated above, since the IFDFW has not yet been validated in Lebanon, validation was performed before moving into validating the entire composite scale.

More specifically, the SES-C score was calculated by summing the codes of the following ten items:

1. The self-declared social class of the participant (poor, lower-middle, upper-middle, and wealthy) (max 4 points);
2. IFDFW categories (8–25; 26–50; 51–75; > 75 points) (max 4 points);
3. Not being in debt (vs. being in debt) (1 point);
4. Household monthly income categories (no income, low, intermediate, and high) (4 points);
5. Work status of the participant (working vs. not) (1 point);
6. Getting no financial help from others (vs. sometimes and regularly) (max 2 points);
7. Crowding index inverse categories (> 3; 1.51–3; 1.1–1.5; 0.51–1; 0–0.5 person/room) (max 5 points);
8. Education level of the head of the family (illiterate, school, and university levels) (3 points);
9. Education level of the participant (illiterate, school, and university levels) (3 points);
10. Work status of the head of the family (working vs. not) (1 point).

Questions with a missing or “prefer not to say” answer were not included in the sum. Before generating the score, items’ codes with inverse directions were reversed to allow for correct scoring and a smoother interpretation of the measured concepts (mainly crowding index categories, being in debt, and getting financial help from others).

The total score ranged from 6 to a maximum of 28. The score was then divided into four 5-point categories, i.e., very low (6–10 points), low (11–16 points), average (17–22 points), and high (23–28 points) socioeconomic categories, regardless of participants’ distribution, for ease of use.

### Statistical analysis

Data were analyzed on SPSS software version 25. A descriptive analysis was performed using absolute frequencies and percentages for categorical variables and means and standard deviations (SD) for quantitative measures.

The construct validity of the IFDFW and SES-C scales was assessed using Principal Component Analysis (PCA), which allows all items to be included, the resulting components to be correlated with the total scale and with each other, and the obtained scores to be directly summed to ensure that the underlying factor structure is appropriate. The Kaiser–Meyer–Olkin (KMO) and Bartlett’s test of sphericity were calculated to ensure the model’s adequacy. Factors with Eigenvalues higher than one were retained, and the scree plot method was used to determine the number of components to extract [24]. Furthermore, items related to SES-C were also correlated to assess convergent validity. The internal consistency of the studied scales was assessed using Cronbach’s alpha: internal consistency values of α ≥ 0.7 and ≥ 0.8 were considered acceptable and excellent, respectively [25]. The Spearman correlation test was used to assess convergent validity by correlating the composite scale and its related items. A p-value less than 0.05 was considered significant.

The normality of continuous variables was checked by the visual inspection of the histogram, while the skewness and kurtosis were below |1.96|. Afterwards, the ANOVA test was used in the bivariate analysis to compare the means of continuous variables between the three SES-C defined categories. Moreover, the percentages derived from dichotomous and multinomial variables were compared using the Chi-square test.

Then, a multiple regression analysis was conducted to decrease potential confounding. Since the dependent variable was categorized (the SES-C scale previously defined categories), a multinomial regression using the backward stepwise method was performed (probability of item in = 0.05; probability of item out = 0.1), taking all the independent variables of the bivariate analysis that are not part of the SES-C scale (gender, age, weight, height, marital status, nationality, cigarette smoking, nargileh smoking, alcohol consumption, chronic disease, easy access to healthcare, governorate, housing condition, and healthcare coverage type). Two models were generated, one for high versus low SES-C category and one for moderate versus low SES-C category. The models’ adequacy to the data was checked before the results were reported. An ordinal logistic regression was also conducted, but the generated model was not adequate to the data (results not reported).

## Results

### Sociodemographic characteristics of the participants

The sociodemographic and other characteristics are displayed in Table 1. Most participants were females (73.4%), single (68.5%), had achieved a university education level (97.3%), were healthcare professionals (70.5%), working (51.8%), with an intermediate or high income (57.6%), and lived in urban areas (72.0%), especially Beirut and Mount Lebanon (74.3%). Most participants reported that the head of the family had a university education level (70.8%) and was working (81.3%). Almost half of the participants belonged to the upper-middle social class (45.8%) and worked full-time (40.2%). Moreover, 14.1% were current cigarette smokers, 20.3% were nargileh smokers, and 26.8% declared consuming alcohol. Also, 17.6% had a chronic disease, and 83.5% had easy access to healthcare. Participants’ mean age ± standard deviation (SD) was 29.52 ± 11.03 years [min = 18; max = 96], and the mean household crowding index was 0.98 ± 0.52.

**Table 1:** Sociodemographic and other characteristics of the participants (*N* = 448)

	**Frequency**	**Percentage**
Gender		
Female	329	73.4
Male	119	26.6
Marital status		
Single	297	66.3
Married	141	31.5
Divorced	10	2.2
Current governorate		
Beirut	148	33.0
Mount Lebanon	185	41.3
North	47	10.5
South	50	11.2
Beqaa	18	4.0
Living place		
Urban	323	72.1
Rural	125	27.9
Work type		
Self-employed/retired/does not work	185	41.3
Full-time	180	40.2
Part-time	83	18.5
Cigarette Smoking status		
No	374	83.5
Yes, current smoker	63	14.1
Yes, previous smoker	11	2.5
Nargileh smoker		
No	342	76.3
Yes, current smoker	91	20.3
Yes, previous smoker	15	3.3
Alcohol consumption		
No	312	69.6
Yes, currently	120	26.8
Yes, previous alcohol consumer	16	3.6
Chronic disease		
Yes	79	17.6
No	369	82.4
Easy access to healthcare		
Yes	374	83.5
No	74	16.5
	**Mean**	**SD**
Age (years)	29.52	11.03
Household crowding index	0.98	0.52
Height in cm	167.29	10.74
Weight in kg	68.03	17.90
Number of children	0.70	1.46

### Validation of the InCharge Financial Distress-Financial Well-Being Scale (IFDFW)

The factor analysis used to assess the construct validity showed that the scale items loaded on one factor; moreover, reliability was excellent, with a Cronbach alpha of 0.914 (Table 2). The mean IFDFW score was 43.06 ± 17.13. After categorization, 78(17.4%) declared belonging to Category 1 (8–25 points), 221(49.3%) to Category 2 (26–50 points), 134(29.9%) to Category 3 (51–75 points), and 15(3.3%) to Category 4 (75–80 points).

**Table 2:** Factor analysis of the IFDFW scale

Factor	Factor 1^a^
**Item 8.** How stressed do you feel about your personal finances in general?	.892
**Item 3.** How do you feel about your current financial situation?	.858
**Item 7.** How frequently do you find yourself just getting by financially and living paycheck to paycheck?	.834
**Item 4.** How often do you worry about being able to meet normal monthly living expenses?	.809
**Item 2.** On the stair steps below, mark (with a circle) how satisfied you are with your present financial situation. The “1” at the bottom of the steps represents complete dissatisfaction. The “10” at the top of the stair steps represents complete satisfaction. The more dissatisfied you are, the lower the number you should circle. The more satisfied you are, the higher the number you should circle	.798
**Item 6.** How often does this happen to you? You want to go out to eat, go to a movie or do something else and don’t go because you can’t afford to?	.788
**Item 5.** How confident are you that you could find the money to pay for a financial emergency that costs about $1,000?	.714
**Item 1.** What do you feel is the level of your financial stress today?	.645
**Percentage variance explained**	63.29%
**Cronbach alpha = 0.914**	
**Kaiser–Meyer–Olkin (KMO) = 0.909**	
**Bartlett’s test of sphericity *p* < 0.001**	

^a^Items are displayed in a descendent order of loading

### Validation of the socioeconomic status composite scale (SES-C)

Table 3 presents the construct validity of the composite scale. The factor analysis yielded a four factors solution after a Varimax rotation; using the PROMAX rotation and trying to fix the number of factors led to similar results. The four factors were overall financial well-being (4 items), social determinants of financial well-being (3 items), education (2 items), and family head work status (1 item).

**Table 3:** Factor analysis of SES-C

Varimax rotated matrix				
**Factor**	**Factor 1**^b^	**Factor 2**^b^	**Factor 3**^b^	**Factor 4**
To which social class do you belong?	.694			
Financial well-being categories	.686			
Not being in debt	.609			
Household monthly income categories	.588			
Work status of the participant (working vs. not)		.781		
Getting no financial help from others		.583		
Crowding Index inverse categories^a^		.435		
Education level of the head of the family			.755	
Education level of the participant			.754	
Work status of the head of the family (working vs. not)				.834
**Percentage variance explained = 53.93%**	19.27	12.33	11.74	10.58
**Kaiser–Meyer–Olkin (KMO) = 0.615**				
**Bartlett’s test of sphericity *p* < 0.001**				

Factor 1: financial wellness; Factor 2: social determinants of financial wellness; Factor 3: education; Factor 4: family head work status

^a^A higher value was assigned a lower code

^b^ Items are displayed in a descendent order of loading

The SES-C score was then calculated by summing the codes of the following items: social class to which the participant belongs (poor, lower-middle, upper-middle, and wealthy), IFDFW categories (8–25; 26–50; 51–75; > 75 points), not being in debt (vs. being in debt), monthly household income (no income, low, intermediate, and high), work status of the participant (working vs. not), getting no financial help from others (vs. sometimes and regularly), crowding index inverse categories (> 3; 1.51–3; 1.1–1.5; 0.51–1; 0–0.5 person/room), family head education level (illiterate, school, and university), participant education level (illiterate, school, and university), and family head work status (working vs. not).

The mean score of SES-C was 21.43 (SD = 2.80), the median was 21.5, and the 25^th^ and 75^th^ percentiles were 19 and 23.5, respectively [IQR = 4.5; min = 14; max = 28]. After categorization, no participants belonged to the very low category, while 22(4.9%), 262(58.6%), and 163(36.5%) were in the low, average, and high categories, respectively. Table 4 describes all the items of the composite scale.

**Table 4:** Description of the composite socioeconomic scale and its items

	**Frequency**	**Percentage**
**Socioeconomic Status Composite scale (SES-C)**		
Very low SES-C category	0	0
Low SES-C category	22	4.9
Average SES-C category	262	58.6
High SES-C category	163	36.5
**Highest achieved education level**		
Illiterate level	2	0.4
School level	10	2.2
University level	436	97.3
**Education level of the head of the family**		
Illiterate level	4	0.9
School level	127	28.3
University level	317	70.8
**Work status**		
Working	216	48.2
Not working	232	51.8
**Work status of the head of the family**		
Working	84	18.8
Not working	364	81.3
**Household monthly income**		
No income	120	26.8
Low income: below 6 million Lebanese Pounds (LBP)	70	15.6
Intermediate income: 6—18 million LBP	115	25.7
High income: over 18 million LBP	143	31.9
**Social class**		
Poor	10	2.2
Lower-middle class	151	33.7
Upper-middle class	205	45.8
Wealthy	8	1.8
I prefer not to answer	74	16.5
**Debt**		
Yes	55	12.3
No	347	77.5
I prefer not to answer	46	10.3
**Financial help**		
Yes sometimes	56	12.5
Yes regularly	28	6.3
No	340	75.9
I prefer not to answer	24	5.4
**Crowding index categories**		
0–0.5 person/room	2	0.4
0.51–1 person/room	45	10.0
1.1–1.5 persons/room	86	19.2
1.51–3 persons/room	243	54.2
More than 3 persons/room	71	15.8
**IFDFW categories**		
0–25 points	78	17.4
26–50 points	221	49.3
51–75 points	134	29.9
> 75 points	15	3.3

Table 5 shows the convergent validity results of the composite scale items. The SES-C scale was correlated with all its items, mostly income, social class, and IFDFW categories, while it was the least but still significantly associated with participant education level and family head work status. IFDFW categories were correlated with many items, while participant education level was only correlated with that of the family head, with a slight correlation with financial well-being. Family head work status was not correlated with any other items.

**Table 5:** Spearman correlation analysis of SES-C items

	**SES-C scale**	Participant education level	Family head education level	Social class	No Debt	No Financial help	Crowding index inverse categories	Participant work status	Family head work status	Monthly Household income categories	IFDFW categories
Participant education level	0.171***	-									
Family head education level	0.330***	0.182***	-								
Social class	0.569***	0.059	0.179***	-							
No debt	0.365***	0.033	0.022	0.267***	-						
No financial help	0.376***	0.058	0.092	0.078	0.140**	-					
Crowding Index inverse categories	0.444***	0.004	0.051	0.076	0.087	0.113*	-				
Participant work status	0.302***	-0.077	0.076	0.032	-0.027	0.149**	0.121*	-			
Family head work status	0.175***	-0.009	0.072	-0.031	0.030	0.057	-0.006	-0.017	-		
Monthly household income categories	0.700***	0.068	0.070	0.347***	0.170***	0.079	0.086	0.163**	0.023	-	
IFDFW categories	0.540***	0.099*	0.046	0.276***	0.198***	0.070	0.156**	-0.046	0.086	0.262***	-

*p*-value *** < 0.001; ** < 0.01; * < 0.05

### Correlates of the SES-C scale

The correlates of the SES-C scale are presented in Table 6. Significant associations were found between socioeconomic status and marital status, alcohol consumption, chronic disease, easy access to healthcare, type of healthcare coverage, age, the number of rooms in the house, and the number of children.

**Table 6:** Bivariate analysis taking SES-C as the dependent variable

	**Composite Socioeconomic Status Scale**			
	**Low SES-C *N* = 22**	**Moderate SES-C *N* = 262**	**High SES-C *N* = 163**	***p*-value**
**Gender**				
Female	16 (4.88%)	183 (55.79%)	129 (39.33%)	0.108
Male	6 (5.04%)	79 (66.39%)	34 (28.57%)	
**Marital status**				
Single	17 (5.74%)	197 (66.55%)	82 (27.70%)	**< 0.001**
Married	4 (2.84%)	60 (42.55%)	77 (54.61%)	
Divorced	1 (10.00%)	5 (50.00%)	4 (40.00%)	
**Nationality**				
Lebanese	22 (5.07%)	252 (58.06%)	160 (36.87%)	0.353
Non-Lebanese	0 (0%)	10 (76.92%)	3 (23.08%)	
**Housing condition**				
Very clean	5 (3.65%)	74 (54.01%)	58 (42.34%)	0.103
Relatively clean	13 (5.02%)	152 (58.69%)	94 (36.29%)	
Relatively dirty	3 (6.52%)	34 (73.91%)	9 (19.57%)	
Very dirty	1 (20.00%)	2 (40.00%)	2 (40.00%)	
**Cigarette smoker**				
No	19 (5.09%)	221 (59.25%)	133 (35.66%)	0.146
Yes, current smoker	1 (1.59%)	37 (58.73%)	25 (39.68%)	
Yes, previous smoker	2 (18.18%)	4 (36.36%)	5 (45.45%)	
**Nargileh smoker**				
No	16 (4.69%)	197 (57.77%)	128 (37.54%)	0.558
Yes, current smoker	6 (6.59%)	57 (62.64%)	28 (30.77%)	
Yes, previous smoker	0 (0%)	8 (53.33%)	7 (46.67%)	
**Alcohol consumption**				
No	19 (6.11%)	190 (61.09%)	102 (32.80%)	**0.048**
Yes, currently	2 (1.67%)	62 (51.67%)	56 (46.67%)	
Yes, previous alcohol consumer	1 (6.25%)	10 (62.50%)	5 (31.25%)	
**Chronic disease**				
Yes	9 (11.39%)	39 (49.37%)	31 (39.24%)	**0.008**
No	13 (3.53%)	223 (60.60%)	132 (35.87%)	
**Easy access to healthcare**				
Yes	13 (3.48%)	210 (56.15%)	151 (40.37%)	**< 0.001**
No	9 (12.33%)	52 (71.23%)	12 (16.44%)	
**Healthcare coverage**				
Private insurance	1 (0.54%)	87 (47.28%)	96 (52.17%)	**< 0.001**
NSSF	6 (7.41%)	46 (56.79%)	29 (35.80%)	
Ministry of Public Health	0 (0%)	2 (66.67%)	1 (33.33%)	
Self-payer	5 (6.25%)	57 (71.25%)	18 (22.50%)	
Public insurance	5 (9.09%)	38 (69.09%)	12 (21.82%)	
Other insurance	5 (11.36%)	32 (72.73%)	7 (15.91%)	
	**Mean ± SD**	**Mean ± SD**	**Mean ± SD**	
Age (years)	24.04 ± 8.56	27.91 ± 9.72	32.89 ± 12.40	**< 0.001**
Weight (Kg)	70.31 ± 26.10	68.15 ± 18.07	67.66 ± 16.27	0.805
Height (cm)	168.72 ± 9.70	168.17 ± 9.59	165.73 ± 12.37	0.061
Number of rooms in the house	3.36 ± 0.95	4.67 ± 1.65	5.60 ± 1.66	**< 0.001**
Number of children	0.77 ± 1.19	0.49 ± 1.23	1.01 ± 1.74	**0.002**

*NSSF* National Social Security Fund

Table 7 presents the multivariable analysis of SES-C categories. Based on the multinomial regression, belonging to the highest SES-C category (versus the lowest) was significantly associated with older age, the absence of chronic disease, and easy access to healthcare. Those in the average SES-C category (versus the low SES-C category) also shared these characteristics, with the added factor of being a former cigarette smoker.

**Table 7:** Multinomial logistic regression

	***p*-value**	**ORa**	**Confidence interval**	
			**Lower Bound**	**Upper Bound**
Model 1: Taking the composite socioeconomic scale (high vs. low*) as the dependent variable				
Marital status (single vs. divorced^a^)	0.207	7.093	0.338	148.850
Marital status (married vs. divorced^a^)	0.395	3.754	0.178	79.133
Cigarette smoker (previous vs. non-smoker^a^)	0.119	0.198	0.026	1.516
Cigarette smoker (current vs. non-smoker^a^)	0.163	5.260	0.511	54.136
**Chronic disease (yes vs. no**^a^**)**	**0.002**	**0.170**	**0.056**	**0.517**
**Easy access to healthcare (yes vs. no**^a^**)**	**0.001**	**7.813**	**2.315**	**26.316**
Health coverage (private insurance vs. self-payer^a^)	0.051	9.874	0.991	98.420
Health coverage (NSSF vs. self-payer^a^)	0.960	0.964	0.232	4.002
Health coverage (public funds vs. self-payer^a^)	0.396	0.554	0.142	2.164
**Age**	**0.011**	**1.134**	**1.029**	**1.250**
Height	0.107	0.957	0.907	1.010
**Model 2: Taking the socioeconomic scale (average vs. low**^a^**) as the dependent variable**				
Marital status (single vs. divorced^a^)	0.113	10.189	0.579	179.388
Marital status (married vs. divorced^a^)	0.504	2.675	0.149	48.108
**Cigarette smoker (previous vs. non-smoker**^a^**)**	**0.011**	**0.078**	**0.011**	**0.563**
Cigarette smoker (current vs. non-smoker^a^)	0.214	4.249	0.433	41.717
**Chronic disease (yes vs. no**^a^**)**	**0.001**	**0.164**	**0.058**	**0.460**
**Easy access to healthcare (yes vs. no**^a^**)**	**0.034**	**3.175**	**1.093**	**9.174**
Health coverage (private insurance vs. self-payer^a^)	0.185	4.612	0.481	44.248
Health coverage (NSSF vs. self-payer^a^)	0.451	0.598	0.157	2.281
Health coverage (public funds vs. self-payer^a^)	0.542	0.680	0.196	2.352
**Age**	**0.022**	**1.119**	**1.016**	**1.232**
Height	0.454	0.981	0.932	1.032

*NSSF* National Social Security Fund

^a^Reference group. The stepwise method was used, and the following variables were included at baseline: age, marital status, current governorate, cigarette smoking, chronic disease, easy access to healthcare, and healthcare coverage type; Weight, height, gender, nationality, governorate, nargileh smoking, alcohol consumption and housing condition were also included, but were removed from the model through the automatic procedure

## Discussion

In this study, a composite scale could be developed and validated based on several aspects of socioeconomic status, i.e., participant education level, family head education level, perceived social class, not being in debt, not receiving financial help, crowding index, participant work status, family head work status, monthly household income, and financial well-being. Consistent with the initial version [22], all eight items of the IFDFW scale loaded on one factor, while the four factors of the composite scale were overall financial well-being (4 items), social determinants of financial well-being (3 items), education (2 items), and family head work status (1 item). These dimensions encompass the four SES dimensions recommended by the American Psychological Association: education, income, employment, and family size/relationships [16]. These dimensions also concur with studies conducted among special populations [10, 26, 27] or those focusing on certain SES aspects [10, 28]. In the Program for International Student Assessment (PISA), the economic, social, and cultural status (ESCS) is used [26, 27]. This complex measure comprises three dimensions, i.e., parental education (PARED), highest parental occupation (HISEI), and household possessions (HOMEPOS), including books in the home, and is intended to be used among schoolchildren. One review suggested improvement to this measure by simplifying and stabilizing how the different components are combined, such as abandoning the use of empirical weights (based on principal component analysis) in favor of arbitrary weights [27]. A study among older individuals used education, social class, occupational complexity, and income to create a composite scale; it found that this composite scale should be preferred in any research addressing health inequalities [10]. Although some studies used education, income, or occupational class to measure SES, these factors cannot be used interchangeably as indicators of a hypothetical latent social dimension as they measure different phenomena and target different causal mechanisms [28]. Nevertheless, to our knowledge, no study has assessed the structural validity and reliability of a comprehensive measurement tool. The composite indicators obtained in our study were assumed to be formative and calculated through a linear combination of variables that did not necessarily share a common concept [29]. From a content perspective, our results confirm those recently published, suggesting items considered most indicative of SES [9]. Thus, the validated composite scale developed in this study can provide a comprehensive assessment of SES by utilizing existing information.

High SES was significantly associated with married status, older age, alcohol consumption, the absence of chronic disease, easy access to healthcare, private insurance coverage, and the number of rooms in the house. The results related to age, chronic disease, and access to healthcare were confirmed in the multivariable analysis comparing high vs. low SES. Participants in the average SES-C category also shared these characteristics, with the added factor of being previous cigarette smokers. The other factors were not significant in the multivariable model, probably due to confounding.

This study showed a strong correlation between high SES and being married in the bivariate analysis. Individuals with high SES are more likely to be married than those with low SES, as they often have more financial stability and, sometimes, better job prospects. Moreover, being married often provides social and economic benefits, such as tax breaks, shared health insurance, and emotional support [30–32]. In our sample, this factor was confounded by age and better access to healthcare. A robust significant association was found between high SES and age. Older individuals tend to have higher SES, as they would have had more time to accumulate wealth and assets and attain higher levels of education and professional success. Those who have been successful in their careers and have achieved financial stability were more likely to be in higher SES categories, which explains our results since most participants were educated and working.

With this in mind, high SES was inversely associated with chronic diseases. A possible explanation would be that people with high SES have access to better healthcare, more nutritious food options, a safer living environment, and more resources and opportunities for physical activity, which helps maintain a healthy lifestyle [33, 34]. Furthermore, participants with higher SES had private insurance coverage, explaining the easy access to healthcare.

Finally, this study found that moderate SES correlated with similar factors as high SES (such as age, absence of chronic disease, and easy access to healthcare), although with a weaker effect (smaller ORa) when low SES was taken as a reference. This finding suggests a dose–effect relationship between SES-C categories and the abovementioned factors, further consolidating our results.

Furthermore, there was no association between smoking status and SES, except for a higher rate of previous smokers among those with a low SES; in Lebanon, resourceless people are regular cigarette and nargileh smokers because of the ease of access to these products and their affordability despite their culminating prices nowadays [35, 36], hence the need to address this problem to promote healthier habits and reduce smoking-related health risks.

### Public health implications

The findings of this study could contribute to the advancement of our understanding of the impact of the socioeconomic crisis in Lebanon, facilitating research and targeted interventions to address disparities and inequalities faced by different socioeconomic groups:

- The relation between SES and age shows the importance of access to education, job opportunities, and financial security in shaping one’s life trajectory and overall well-being.
- The absence of an association between smoking status and SES, even among resourceless people, is likely due to the fact that smoking in Lebanon is common, affordable, and accessible to all age categories and social classes [35, 36]. This finding has far-reaching implications for public health, as excessive smoking has been linked to various health problems, such as lung disease, cancers, and cardiovascular diseases. Hence, it is essential to address this problem to promote healthier habits and reduce smoking-related health risks
- In terms of access to health, addressing inequalities in SES and providing access to adequate resources and health services can significantly impact public health and reduce the burden of chronic diseases.

This composite scale could be used by researchers, policymakers, and practitioners to measure the socioeconomic status of individuals and communities and inform evidence-based decision-making to improve the overall well-being of the population in a socioeconomic crisis context.

### Limitations

This study has several limitations. Its cross-sectional design only provides a snapshot in time, which limits the ability to establish causality or make inferences about changes over time. There is also a possibility for recall bias or social desirability bias due to the use of self-declared measures for data collection, where participants may not accurately report their behaviors, attitudes, or experiences. Additionally, selection bias might have occurred, as most participants were young and educated, and individuals with limited digital literacy may have been excluded. However, the survey was available in both Arabic and English, the native language and commonly used language in Lebanon, and most residents in the country possess smartphones and are familiar with online access, leading to the belief that this bias was minimal. The sampling bias can also be due to the snowball sampling method, which relies on participants recruiting others from their social networks and can introduce biases, as participants may be more likely to recruit individuals with similar characteristics or experiences. Due to the lack of sampling frames in Lebanon, the non-probability sampling was necessary and could have led to a lower statistical power, which would need to be later corrected through studies with random or larger samples. Furthermore, residual confounding might have occurred, although all potential confounders were taken into account. In addition, although exploring different methods gave similar results, some components of the factor analysis had less than three items, which would lead us to interpret the factor analysis results with caution. Hence, our results should be interpreted with caution and confirmed by further studies.

## Conclusion

In conclusion, the study successfully developed and validated a composite scale for measuring the socioeconomic status in Lebanon, taking into account the complexities of the Lebanese context. The scale was found to be reliable and valid, and its results showed significant correlations with various factors, such as older age, less chronic disease, and easy access to healthcare. Further studies among people from different age groups are necessary to confirm our findings.

## Data Availability

The datasets generated during and/or analyzed during the current study are available from the corresponding author on reasonable request.

## Abbreviation

SES-C: Socioeconomic Status Composite Scale
SES: Socioeconomic status
ORa: Adjusted Odds Ratio
IFDFW: InCharge Financial Distress-Financial Well-Being Scale
SPSS: Statistical Package for the Social Sciences
KMO: Kaiser–Meyer–Olkin
SD: Standard deviation
PCA: Principal component analysis
LBP: Lebanese Pounds
NSSF: National Social Security Fund
